# Host-microbiota interactions: The aryl hydrocarbon receptor in the acute and chronic phases of cerebral ischemia

**DOI:** 10.3389/fimmu.2022.967300

**Published:** 2022-08-12

**Authors:** Xuemei Fan, Shuai Wang, Shuqi Hu, Bingjie Yang, Hao Zhang

**Affiliations:** ^1^ Department of Neurology, Affiliated Hangzhou First People’s Hospital, Zhejiang University School of Medicine, Hangzhou, China; ^2^ Department of Intensive Care Medicine, Affiliated Hangzhou First People’s Hospital, Zhejiang University School of Medicine, Hangzhou, China

**Keywords:** aryl hydrocarbon receptor, microbiota-gut-brain axis, cerebral ischemia, tryptophan metabolism, gut microbiota

## Abstract

The relationship between gut microbiota and brain function has been studied intensively in recent years, and gut microbiota has been linked to a couple of neurological disorders including stroke. There are multiple studies linking gut microbiota to stroke in the “microbiota-gut-brain” axis. The aryl hydrocarbon receptor (AHR) is an important mediator of acute ischemic damage and can result in subsequent neuroinflammation. AHR can affect these responses by sensing microbiota metabolites especially tryptophan metabolites and is engaged in the regulation of acute ischemic brain injury and chronic neuroinflammation after stroke. As an important regulator in the “microbiota-gut-brain” axis, AHR has the potential to be used as a new therapeutic target for ischemic stroke treatment. In this review, we discuss the research progress on AHR regarding its role in ischemic stroke and prospects to be used as a therapeutic target for ischemic stroke treatment, aiming to provide a potential direction for the development of new treatments for ischemic stroke.

## Introduction

Cerebrovascular accident, commonly known as stroke and being a global health concern, is characterized by high mortality and disability rates, and is one of the leading cause of dementia and depression (1). According to neuropathology, stroke can be classified into two major subtyes: ischemic and hemorrhagic, with the former and latter accounting for 85% and 15% of all cases, respectively (2). The relationship between gut microbiota and brain function has been studied intensively in recent years, and gut microbiota has been linked to a couple of neurological disorders, including Alzheimer’s disease (AD) (3), Parkinson’s disease (PD) (4), multiple scleroses (MS) (5), neurodevelopmental (6) and psychiatric disorders (7, 8), and stroke (9–15). Communication between the brain and gut microbiota is mainly mediated by neurogenic signaling molecules and microbial metabolites; specifically, four pathways related to neuro, metabolism, endocrine, and immune signaling, are involved in this process (16). In turn, the central nervous system (CNS) can regulate neurotransmitters to achieve bidirectional communications by shaping microbial community composition and function. These processes that link microbiota and the brain are termed the “microbiota-gut-brain” axis. Study have proven that the gut microbiota can influence stroke prognosis by modulating the immune response and neuroinflammation (13). In turn, stroke can induce a shift in the gut microbiota, affecting intestinal motility and permeability, stress response, and systemic infection after stroke (10, 14, 15, 17). These findings highlight the close connection between gut microbiota and stroke in the “microbiota-gut-brain” axis.

Gut microbiota interacts with the host mainly through its metabolites. Tryptophan is an essential amino-acid that must be obtained from the diet. It can be metabolized by gut microbiota directly or indirectly and participates in a variety of physiological processes. Abnormal tryptophan metabolism has been associated with many diseases. The AHR is an important mediator of acute ischemic damage and can result in subsequent neuroinflammation (18, 19). AHR can affect these responses by sensing microbiota metabolites. For instance, it can be activated predominantly by ligands produced from gut microbes metabolizing diet-derived tryptophan (20, 21). Indeed, aberrant tryptophan metabolism and dysbiosis of gut microbiota have been observed in both acute and chronic stages of cerebral ischemia (22, 23). Actually, ischemic injuries and subsequent neuroinflammation have been recognized as key elements in stroke development. Neuroinflammation exists in both acute and chronic phases of cerebral ischemia, affecting the prognosis and survival of stroke patients to some extent. Persistent neuroinflammation could induce neurodegeneration, leading to post-stroke dementia and depression (24, 25). Activated microglia and astrocyte play an important role in neuroinflammation after stroke, which may be achieved through the binding of the ligand to AHR (5, 26–30).

AHR is engaged in the regulation of acute ischemic brain injury and may be involved in chronic neuroinflammation after stroke. As an important regulator in the “microbiota-gut-brain” axis, AHR has the potential to be used as a new therapeutic target for ischemia stroke treatment. In this review, we discuss the research progress on AHR regarding its role in ischemia stroke and prospects to be used as a therapeutic target for ischemia stroke treatment, aiming to provide a potential direction for the development of new treatments for ischemia stroke.

## Role of the “microbiota-gut-brain” axis in the development of ischemia stroke

**Figure 1:**
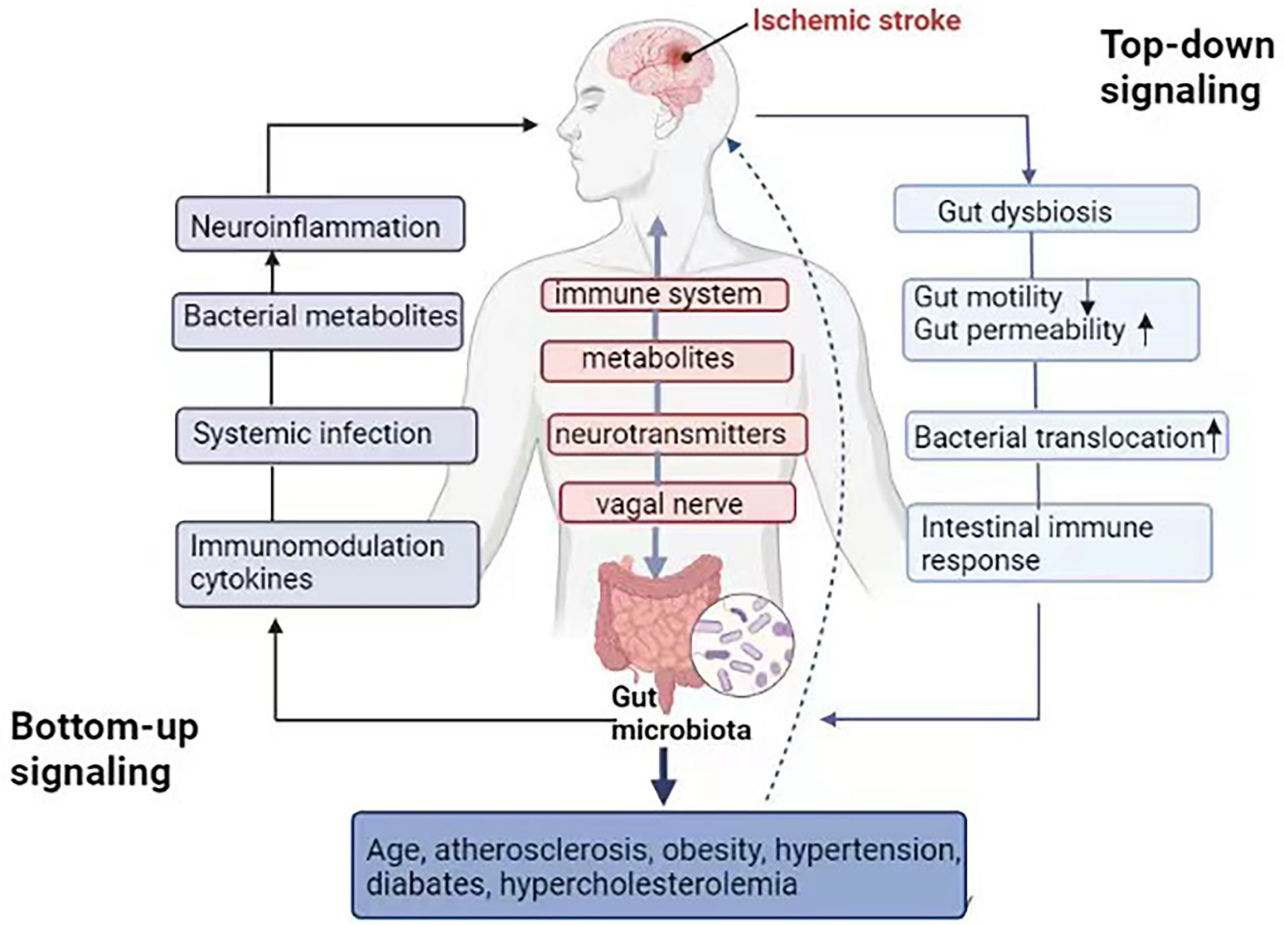
“Microbiota-gut-brain” axis in the ischemic stroke. Gut microbiota communicates to the CNS through the immune system, metabolites and neurotransmitters, as well as activation of the vagal nerve. In the top-down signaling pathway, ischemic stroke can affect the community structure and function of the gut microbiota through the autonomic nervous system, increase the gut permeability and reduce `gut motility, meanwhile, inducing an intestinal immune response and bacterial translocation. In the bottom-up signaling pathway, gut microbiota dysbiosis after stroke leads to changes in bacterial metabolites, systematic infection due to bacterial translocation, immune cell migration and the release of immunomodulation cytokines, which further mediate neuroinflammation, related to the severity of ischemic stroke and worse prognosis. Gut microbiota can affect risk factors related to ischemia stroke directly or indirectly, including hypertension, diabetes, hypercholesterolemia, obesity and atherosclerosis, as well as aging.

The communication between the gut microbiota and CNS is mediated through at least 4 interacting components, including the immune system, metabolites, neurotransmitters, and activated vagal nerve (19). In the top-down signaling pathway, ischemia stroke can disrupt the structure and function of the gut microbiota through the autonomic nervous system, increasing the gut permeability and reducing its motility, which further induces an intestinal immune response and bacterial translocation. In the bottom-up signaling pathway, post-stroke gut microbiota dysbiosis can result in changes in bacterial metabolites, leading to systematic infection due to bacterial translocation, abnormal immune cell migration, and release of immunomodulatory cytokines, which further mediates neuroinflammation that causes severe ischemia stroke and worse prognosis (31) (**Figure 1**).

Preclinical and clinical studies demonstrated that gut microbiota plays an important role in the pathogenesis and prognosis of ischemia stroke (10, 12–16, 32–47) (**Table 1**). For example, many studies indicate that gut microbiota can affect risk factors related to ischemia stroke directly or indirectly, including hypertension, diabetes, hypercholesterolemia, obesity and atherosclerosis, as well as aging (35, 48–52). However, so far, there is no large prospective study exploring how gut microbiota relates to the long-term risk of ischemia stroke. In addition, ischemia stroke could change the gut microbiota composition. For instance, *Enterobacteriaceae*, *Ruminococcaceae*, *Veillonellaceae* and *Lachnospiracea*e were significantly enriched after stroke, while *Bacteroidaceae* and *Prevotellaceae* were significantly reduced. *Enterobacteriaceae* showed notably increased in patients with poor prognosis of cerebral infarction (47). Another study demonstrated that dysbiosis of the gut microbiota relates to ischemia stroke severity in mice; specifically, germ-free (GF) mice can develop more severe brain injury after receiving fecal transplants from high-stroke disequilibrium index (SDI) mice (36). Pre-existing microbiota ensures intestinal protection, and transplantation of the gut microbiota from post-stroke mice to GF mice exacerbates the brain damage and functional deficits compared to those in the controls. GF mice present enlarged brain lesions compared to recolonized (Ex-GF) and specific pathogen-free (SPF) mice after stroke (11). Changes in gut microbiota induced by antibiotics such as ampicillin can reduce ischemic brain injury and gut inflammation, leading to improved long-term prognosis (16, 38). However, another study showed the opposite result; mortality in mice with disturbed gut microbiota was significantly higher following inhibition of gut microbiota by broad-spectrum antibiotics (12). Therapeutic fecal microbiota transplantation (FMT) can normalize the microbiota imbalance induced by brain injury and improve stroke prognosis (10). This effect may be particularly pronounced when aged stroke mice received FMT from young mice. The aged mice showed fewer behavioral abnormalities and neuroinflammation, which may be due to the fact that gut microbiota could produce high levels of short-chainfatty acids (SCFAs). Mechanistically, SCFAs can improve neuronal connectivity and synaptic plasticity after stroke by modulating microglia activation through recruitment of T-lymphocytes, thereby improving behavioral recovery. Studies have shown that supplementation of *Lactobacilli* after stroke can reduce neuroinflammation and improve cognitive function and depression (35, 40, 45). In addition, new evidence indicates that lactulose and atorvastatin may regulate the structure of gut microbiota by regulating intestinal immune function and reducing neuroinflammation after stroke (43, 46).

**Table 1:** Summarizes the pre-clinical and clinical evidences regarding the relationship between gut microbiota and ischemic stroke.

Author	Year of publication	Type of study	Subjects	Key findings
Caso et al. (32)	2009	Pre-clinical study	CCAO and MCAO rat	Bacterial translocation to mesenteric lymph nodes, spleen, liver, and lung after stroke, and it was associated with worsening stroke.
Benakis et al. (16)	2016	Pre-clinical study	MCAO mice	Antibiotic-induced alterations in the gut microbiota can reduce ischemic brain injury, the effect can be transmitted by FMT.
Singh et al. (10)	2016	Pre-clinical study	MCAO mice	Reduced species diversity and bacterial overgrowth of bacteroidetes were associated with intestinal barrier dysfunction and reduced intestinal motility; gut dysbiosis intensifies the ingress of Th17- and IL17-secreting γ δ T-cells (γ δ T-cells) into the CNS from the intestine, leading to chronic systemic and neuroinflammation. Higher numbers of proinflammatory lymphocyte populations correlate negatively with stroke outcome, which is reflected as larger infarct size, brain edema, and neurological deficits; FMT improves stroke outcome.
Houlden et al. (15)	2016	Pre-clinical study	MCAO mice	Specific changes in Peptococcaceae and Prevotellaceae were related with the severity of the stroke; changes in gut microbiota after stroke may affect recovery and treatment.Gut dysbiosis affects the local immune cells in the intestine and brain. In the early stage of stroke, engages both innate and adaptive immunity, microglial activation is followed by infiltration of peripheral immune cells, including monocytes, T- and B-lymphocytes.
Winek et al. (12)	2016	Pre-clinical study	MCAO mice	Conventional microbiota ensures intestinal protection; microbial colonization or specific microbiota are crucial for stroke outcome.
Stanley et al. (14)	2016	Pre-clinical study	MCAO mice	Stroke promotes the translocation and dissemination of selective strains of bacteria that originated from the host gut microbiota.
Crapser et al. (33)	2016	Pre-clinical study	MCAO mice	Ischemic stroke induces gut permeability and enhances bacterial translocation leading to sepsis in aged mice.
Yamashiro et al. (34)	2017	Clinical study	41 patients: 40 controls	Ischemic stroke was independently associated with increased bacterial counts of Atopobium cluster and Lactobacillus ruminis, and decreased numbers of Lactobacillus sakei subgroup, changes in the prevalence of Lactobacillus ruminis were positively correlated with serum IL-6 levels.
Spychala et al. (35)	2018	Pre-clinical study	MCAO mice	The Firmicutes to Bacteroidetes ratio in aged mice increased 9-fold compared to young; gut microbiota can be modified to positively impact outcomes from age-related diseases.
Singh et al. (11)	2018	Pre-clinical study	MCAO mice	Bacterial colonization reduces stroke volumes by increasing cerebral expression of cytokines and microglia/macrophage cell counts; lymphocyte-driven protective neuroinflammation after stroke under control of the microbiome.
Xia et al. (36)	2019	Clinical study	83 patients: 70 controls	Dysbiosis of the gut microbiota correlated with ischaemic stroke severity, mice receiving FMT from patients with a high stroke disequilibrium index (SDI) developed more severe brain damage
Chen et al. (23)	2019	Pre-clinical study	MCAO cynomolgus monkeys.	The levels of Bacteroidetes phylum and Prevotella genus were significantly increased, the Firmicutes phylum, the Faecalibacterium, Oscillospira, and Lactobacillus genera were decreased after cerebral infarction in monkeys; Cerebral infarction induces persistent host gut microbiota dysbiosis, intestinal mucosal damage, and chronic systemic inflammation in cynomolgus monkeys.
Zeng et al. (37)	2019	Clinical study	141 patients	Compared with the low-risk group, opportunistic pathogens (Enterobacteriaceae and Veillonellaceae) and lactate-producing bacteria (Bifidobacterium and Lactobacillus) were increased, butyrate-producing bacteria (Lachnospiraceae and Ruminococcaceae) were decreased in the high-risk group.
Benakis et al. (38)	2020	Pre-clinical study	MCAO mice	Bacteroidetes S24.7 and the enzymatic pathway for aromatic metabolism were correlated with infarct volume; The gut microbiota composition in the ampicillin-treated mice was associated with reduced gut inflammation, a long-term favorable outcome, and a reduction of brain tissue loss.
JeoJeonn et al. (39)	2020	Pre-clinical study	MCAO pig	Abundance of the Proteobacteria was significantly increased, while Firmicutes decreased at 3 days poststroke, compared to prestroke populations, abundance of the lactic acid bacteria Lactobacillus was reduced. By day 5, the microbial pattern returned to similar values as prestroke,
Lee et al. (40)	2020	Pre-clinical study	MCAO mice	Aged stroke mice receiving young fecal transplant had less behavioral impairment and inflammation, which is related to Bifidobacterium longum, Clostridium symbiosum, Faecalibacterium prausnitzii and Lactobacillus fermentum, for they can produce more SCFAs
Ling et al. (41)	2020	Clinical study	93 patients	The abundance of Firmicutes and its members, including Clostridia, Clostridiales, Lachnospiraceae, and Lachnospiraceae_other, was significantly decreased in the age-matched PSCI group; PSCI was significantly correlated with the abundance of Enterobacteriaceae after adjustments
Xiang et al. (42)	2020	Clinical study	20 patients: 16 controls	Stroke patients had fewer Firmicutes than controls. Lachnospiraceae (OTU_45) and Bacteroides served as markers of lacunar infarction. Bilophila and Lachnospiraceae (OTU_338), served as markers of non-lacunar acute ischemic infarction. Three optimal bacterial species, Pseudomonas.
Yuan et al. (43)	2021	Pre-clinical study	MCAO mice	Lactulose promotes functional outcomes after stroke in mice, which may be attributable to repressing harmful bacteria, and metabolic disorder, repairing gut barrier disruption, and reducing inflammatory reactions after stroke.
Wu et al. (44)	2021	Pre-clinical study	MCAO rat	The abundance of the Firmicutes phylum was decreased, whereas Proteobacteria and Deferribacteres were increased after stroke; Ruminococcus_sp_15975 might serve as a biomarker for the stroke; Many metabolites, such as L-leucine, L-valine, and L-phenylalanine, differed between the stroke and sham groups
Huang et al. (45)	2021	Pre-clinical study	MCAO mice	Bifidobacterium was enriched in calorie-restriction mice; Bifidobacterium administration improved the long-term rehabilitation of stroke mice
Zhang et al. (46)	2021	Pre-clinical study	MCAO mice	Atorvastatin increased the abundance of Firmicutes and Lactobacillus, decreased Bacteroidetes abundance, increased fecal butyrate level, promoted intestinal barrier function, regulated intestinal immune function, and reduced microglia-mediated neuroinflammation after stroke; FMT of atorvastatin-treated mice alleviated neuroinflammation in MCAO mice.
Xu et al. (47)	2021	Clinical study/Pre-clinical study	28patients: 28controls	Enterobacteriaceae, Ruminococcaceae, Veillonellaceae and Lachnospiraceae were significantly enriched after stroke, while Bacteroidaceae and Prevotellaceae were significantly reduced. Enterobacteriaceae showed significant enrichment in patients with poor prognosis of cerebral infarction. Enterobacteriaceae exacerbates cerebral infarction by accelerating systemic inflammation and alleviates cerebral infarction by inhibiting its excessive growth.

CCAO, common carotid artery occlusion.

Acute ischemia stroke is characterized by loss of species diversity and overgrowth of opportunistic pathogens. A previous study has shown that acute ischemia stroke patients can develop significant gut microbiota disturbances at 3 days post-stroke, which returned to similar levels pre-stroke by day 5 (39). However, cerebral ischemia can induce persistent gut microbiota dysbiosis, disrupt the gut barrier, and lead to chronic systemic inflammation of the host, which is associated with worsening stroke and neurodegenerations. One study demonstrated that gut dysbiosis could last for more than 3 weeks after stroke, and then the disturbed gut microbiota could gradually recover but microbiota diversity was still decreased significantly after 4 weeks (36). Gut microbiota dysbiosis in Cynomolgus monkeys is still observed 6 and 12 months after cerebral ischemia, with notably increased *Bacteroidetes phylum* and *Prevotella* genus and significantly reduced *Firmicutes phylum*, *Faecalibacterium*, *Oscillospira*, and *Lactobacillus* genera, accompanied by a significant increase in levels of plasma D-lactate, zonulin, LPS, TNF-α, IFN-γ, IL-6 and a significant decrease in levels of SCFAs (23).

## Alterations of levels of tryptophan metabolites and AHR after ischemia stroke

Tryptophan metabolism in the gastrointestinal tract can be regulated by three main pathways, i.e., the kynurenine pathway, serotonin pathway, and indol pathway. Approximately 90% of ingested tryptophan is degraded through the kynurenine pathway in immune and epithelial cells (53, 54). More specifically, tryptophan is transferred into the brain crossing the blood-brain barrier (BBB); then, two key enzymes in the kynurenine pathway, indoleamine-2,3-dioxygenase (IDO) and tryptophan-2,3-dioxygenase (TDO), metabolize L-tryptophan into L-Kyn (55, 56), which plays a key role in this pathway. L-Kyn can be further catabolized in two types of cells, astrocytes and microglia, in the brain. In astrocytes, L-Kyn was transformed into kynurenic acid (KYNA) under the catalyzation of kynurenine aminotransferase (KAT) family enzymes. KYNA is a well-recognized N-methyl-D-aspartate receptor (NMDAR) antagonist and is thought to be neuroprotective (57). In microglia, L-Kyn can be hydroxylated by kynurenine 3-monooxygenase (KMO) to generate 3-HK and its major metabolites, such as quinolinic acid (QUIN), which is considered to be an NMDAR agonist with neurotoxic properties (58). Both QUIN and KYNA act on NMDAR in the postsynaptic membrane of neurons. L-Kyn is shown to be a key AHR ligand and is associated with ischemia stroke severity and prognosis (18).

Preclinical and clinical studies have shown altered kynurenine pathway and tryptophan catabolism after cerebral ischemia. An increased level of brain QUIN was observed in gerbils, which was mediated by the activation of IDO, KYN, and 3-HK after transient ischemic attack (TIA), ultimately leading to an abnormal increase in the QUIN/KYNA ratio, which might contribute to the progression of post-stroke injury (59–63). QUIN is primarily detected in microglia and infiltrating macrophages 2-7 days after cerebral ischemia, which is consistent with a peak in immune infiltration, glial activation and inflammation during this period (62). An altered kynurenine pathway metabolism was observed in a permanent middle cerebral artery occlusion (MCAO) mouse model after ischemia stroke (22). The level of L-Kyn was increased in the brain as early as 3 hours after MCAO and remained at an increased level for 24 hours, in contrast to a decrease in L-tryptophan level between 3 and 24 hours and slight changes in plasma L-Kyn or L-Trp. An increase in AHR protein level, nuclear translocation and transcriptional activity of cortical neurons in this mouse model was also observed. In addition, the L-Kyn/L-Trp ratio is much higher in stroke patients than that in healthy controls and is positively correlated with infarct volume (63). The most common long-term complications after ischemia stroke are dementia and depression. A study found that abnormal alterations in kynurenine pathway catabolism persisted for at least 1 year after stroke, suggesting that it might be the cause of persistent brain dysfunction in these patients (64). The association between cognitive impairment and the kynurenine pathway after ischemia stroke has been described in only one study (65). Decreased levels of 5-HT and increased levels of kynurenine pathway catabolites have been reported in post-stroke patients with depression, and activation of key enzymes in the kynurenine pathway can lead to increased production of 3-HK, QUIN, L-Kyn, and KYNA (66), which induce the production of neurotoxic agents (67, 68). Finally, these alterations can cause damages to multiple brain regions such as the hippocampus, inhibiting neurogenesis and activating apoptotic signaling pathways, and thus leading to depression (69), which has been referred to as the kynurenine hypothesis of depression (70). However, no correlations between depressive symptoms in post-stroke populations and blood L-Kyn/L-Trp ratios have been found (71, 72).

## Gut microbiota affects levels of tryptophan metabolites and AHR

Gut microbes can metabolize tryptophan through several metabolic pathways and produce various tryptophan metabolites (73). For instance, some bacterial species, such as *Escherichia coli*, *Clostridium* spp. *Bacteroides* spp. *Clostridium sporogenes*, *Peptostreptococcus* spp. *Peptostreptococcus russellii*, *Peptostreptococcus anaerobius* and *Peptostreptococcus stomatis*, *Clostridium botulinum*, and *Peptostreptococcus anaerobius*, can produce indole propionic acid (IPA), indoleacetic acid (ILA), and indoleacetic acid (IA). While other species, such as *Lactobacilli*, *Ruminococcus gnavus*, *Clostridium bartlettii*, and *Bifidobacterium* spp., can produce indole aldehydes (IAld), indoleacetic acid (IAA), and ILA. Some others, such as *Bacteroides* spp. and *Clostridium* spp., can produce 3-methylindole (skatole) by decarboxylation of IAA.

Gut microbiota can directly or indirectly metabolize tryptophan, generating various metabolites, including indole, tryptamine, indole ethanol (IE), IPA, ILA, IAA, skatole, IAld and IA. Some of them, including Indole, IPA, and IA, can reduce intestinal permeability by disrupting mucosal homeostasis. Several other tryptophan catabolic products can regulate innate and adaptive immune responses by binding to AHR in intestinal immune cells. For example, IAld can increase IL-22 production by activating the AHR signaling pathway. Some other tryptophan metabolites, such as IPA, IE and IA, can be absorbed into the blood through the intestinal epithelium and exert antioxidant and anti-inflammatory effects (73). Tryptophan catabolic products, including IAA, IA, IAld, ILA, tryptamine, and skatole, are all ligands for AHR (74–77). Some agonists can facilitate AHR in crossing the BBB. In astrocytes and microglia, AHR can inhibit pro-inflammatory nuclear factor-κB (NF-κB) signaling, thus interfering chemokine production and transcriptional programs associated with inflammatory monocyte recruitment, and activating CNS-resident myeloid cells and producing direct neurotoxicity to regulate CNS inflammation (5).

## The role of AHR in ischemia stroke

### The basic characteristics of AHR

AHR is a ligand-controlled transcription factor (5), which is implicated in multiple physiological and pathological processes of many diseases, including inflammatory bowel disease (78), metabolic syndrome, and CNS diseases (79, 80). Expression of AHR is widely detected in the CNS, such as in neurons, oligodendrocytes, monocytes/macrophages, astrocytes, microglia, and cerebral endothelial cells (81). AHR can regulate the expressions of target genes which relate to cell proliferation, metabolism and immune response (82). Significant upregulation of AHR expression after stroke has been reported, which is shown to play a role in the cerebral ischemic injury (22, 83–86) (**Table 2**). In addition, the integrity of the BBB is also compromised upon activation of the AHR signaling (87–89). The BBB is essential for maintaining CNS homeostasis, and impairment of BBB is thought to contribute to neurodegeneration, leading cognitive impairment in humans (90).

**Table 2:** A summary of the role of AHR in cerebral ischemia.

Reference	Subjects	Key findings	Moechanism
Cuartero et al., 2014 (22)	MCAO mice	Ischemic insult increases total and nuclear AHR levels as well as AHR transcriptional activity in neurons *in vivo* and *in vitro*, increasing infarct size and neurological deficits. L-kynurenine-AHR pathway mediates acute brain damage after stroke.	L-Kyn increased the expression of the AHR target genes Cyp1a1 and Cyp1b1 mRNA in cortical neurons; L-Kyn decreased CRE-mediated transcription in neurons, demonstrated by a reduction in both BDNF and NPAS4 mRNA expression to increase apoptosis.
Chen et al., 2019 (83)	MCAO mice	The kynurenine/AHR activation mediated acute ischemic injury. Compared to normal WT controls and AHRcKO mice. AHR immunoreactivities were increased predominantly in activated microglia and astrocytes, leading to a significantly aggrandized ischemic brain infarction, sensorimotor deficits, and nonspatial working memory after MCAO.	AHR affected pro-inflammatory cytokines IL-1β, IL-6, IFN-γ, CXCL1, as well as S100β, NGN2, and NGN1 gene and protein expression after MCAO. TMF treatment modulated gene and protein expression related to neurogenesis after stroke, leading an increased proliferation of neural progenitor cells at the ipsilesional neurogenic zones.
Kwon et al., 2020 (84)	TMCAO rat	The inhibition of AHR activation before reperfusion alleviates brain damage due to apoptosis. AHR antagonism at a delayed time point after ischaemia is also effective in suppressing cerebral I/R injury and this effect was most pronounced in the 10 min and 50 min post-stroke administration groups.	AHR antagonists after ischaemia affected the inhibition of the formation of cellular and vasogenic oedemas due to cerebral I/R.
Tanaka et al., 2021 (85)	MCAO mice	MCAO upregulated AHR expression in microglia during ischemia. MCAO increased the expression of TNFα and then induced edema progression, and worsened the modified neurological severity scores, with these being suppressed by administration of an AHR antagonist, CH223191.	In MCAO model mice, the NOX subunit p47phox expression was upregulated in microglia by ischemia, aggrandized the expression of Tnfa and edema progression. AHR antagonist can relieve hypoxia/ischemia and edema progression and improve the neurological severity scores in mice *via* inhibition of the AHR signaling pathway.
Rzemieniec et al., 2019 (86)	mice	A selective AHR modulator, DIM protects neurons against ischemia-induced damage at earlier and later stages of neuronal development,	Ischemia-induced apoptosis and autophagy and possibly corresponds to ischemia-evoked disruption of HDAC activity and AhR/CYP1A1 signaling pathway. DIM partially reversed OGD-induced apoptosis, autophagy and AHR/CYP1A1 signaling as well as OGD-inhibited HDAC activity.

OGD, Oxygen and glucose deprivation; DIM, 3,3′-diindolylmethane; TNFα, tumor necrosis factor α; NOX, NADPH oxidase; TMCAO, transient middle cerebral artery occlusion.

The role of AHR in the neurological and immune systems has received increasing attention (91). The role of neuroinflammation in acute and chronic ischemia stroke has also been recognized (92). In fact, one of the pathological features of neurodegenerative diseases is neuroinflammation, mainly manifested by chronic activation of microglia (93). AHR can mediates inflammatory effects of microglia through dietary and microbial metabolites, particularly tryptophan metabolites (5, 93). Given the links between tryptophan metabolism, AHR and immune cell activation (94), we will highlight the role of the AHR signaling pathway (i.e., tryptophan metabolites as AHR ligand can bind to AHR) in ischemia stroke and potential targets for pharmacological modulation of ischemia stroke, in the following discussions.

### AHR in acute phases of ischemia stroke

Cuartero et al. used mouse models to verify the hypothesis that activation of the L-Kyn-AHR signaling pathway can exacerbate acute ischemic brain injury (22). They identified increased AHR protein level, nuclear translocation and transcriptional activity of cortical neurons in a permanent MCAO mouse model. In the core of the infarct, the AHR protein level rose to a peak at around 5 hours after stroke and returned to baseline levels by day 7 after stroke; in the peri-infarct area, the AHR protein level started to increase at 18 hours after stroke and reached the peak at day 3 after stroke and then started to decrease. Treating with an AHR antagonist or using AHR-deficient mice resulted in a smaller infarct size and lower National Institutes of Health Stroke Scale (NIHSS) in mice model (22). However, another group showed an opposite result when treating ischemia stroke using the AHR agonists. Mechanistically, activation of the AHR signaling during cerebral ischemia may mediate specific pathological effects by inhibiting the cAMP response element-binding protein (CREB) signaling pathway. Further experiments demonstrated that L-Kyn could accumulate in the brain during acute ischemia stroke and act as an endogenous activator of AHR. Exogenous supplementation of L-Kyn aggravates strokes in an AHR-dependent manner and increases infarct volume. Most interestingly, the authors also demonstrated that inhibition of L-Kyn production by pharmacological blockade of TDO could decrease the activation of AHR signaling and reduce infarct volume in the MCAO stroke model. Taken together, this study identified the L-Kyn-AHR pathway as a novel mediator of brain injury during stroke, and validated TDO and AHR as new “druggable” targets for acute ischemia stroke.

Another study suggested that AHR inhibition in acute ischemia stroke might be benefits regarding functional outcomes through reducing pro-inflammatory glial cell proliferation and promoting neurogenesis. Compared to respective controls, wild-type (WT) and AHRcKO mice that were treated with the AHR antagonist, 6,2’,4’-trimethoxyflavone (TMF), showed significantly smaller infarct volumes and improved sensorimotor and non-spatial working memory functions. AHR Immunoreactivity was increased mainly in activated microglia and astrocytes after AHRcKO. TMF-treated WT and AHRcKO mice showed remarkably increased astrocyte and microglia proliferation (28). In a cerebral ischemia-reperfusion injury (CIRI) rat model, TMF-treated rats displayed lower cell apoptosis levels and smaller infarct volumes than those not treated with TMF at 24 h after cerebral ischemia, which were most pronounced in the 10 min and 50 min after stroke. This study indicated that the AHR antagonists might reduce CIRI-related cellular injury.

### AHR in chronic ischemia stroke

Ischemia stroke can induce long-term host gut microbiota dysbiosis, impairing the intestinal barrier and leading to chronic neuroinflammation. This inflammatory response is associated with cognitive impairment, depression, and anxiety in post-stroke patients (28). One year after FMT, elevated plasma pro-inflammatory cytokines, such as IFN-g, IL-6 and TNF-α, were decreased in focal cerebral ischemia of monkey models, suggesting the persistence of systemic inflammation post ischemia stroke (23). Numerous studies have shown that resident inflammatory cells and microglia can first respond to CIRI and amplify neuroinflammation by interacting with astrocytes (95–98). The inflammatory response in the brain of rats surviving 2 years after ischemic brain injury was evident but varied in the extent regarding microglia and astrocyte responses in different brain tissues (25). In another rat model of dementia in which the rats survived 2 years after cerebral ischemia, it was shown that this neuroinflammatory process was mainly regulated by microglia and astrocyte activity. In conclusion, microglia and astrocytes play an important role in post-stroke neurodegeneration (99).

**Figure 2:**
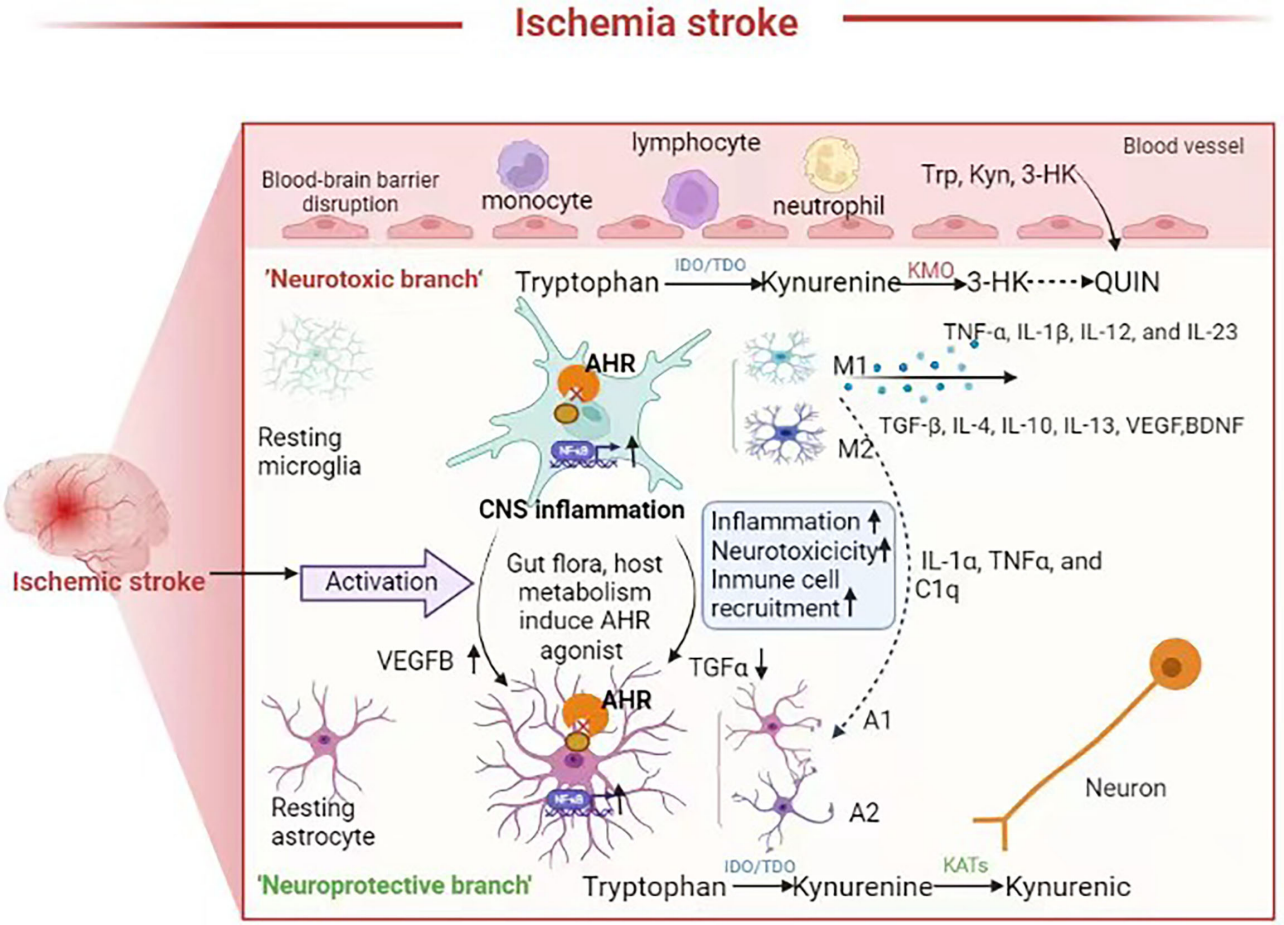
Neuroinflammation in the brain after stroke, the role of AHR and tryptophan metabolites in neuroinflammation. Microglia and astrocytes are activated and interact with each other to mediate neuroinflammation following ischemic stroke Some metabolites such as 3-HK, Kyn, QUIN produced by tryptophan metabolism can cross the BBB plays a neuroprotective or neurotoxic role. Gut flora and the host tryptophan metabolism produce AHR agonist. In astrocytes and microglia, AHR can inhibit pro-inflammatory nuclear factor-κB (NF-κB) signaling, and reduction of AHR agonists after gut microbiota dysbiosis result in an upregulated neuroinflammation and neurotoxic responses and immune cell recruitment, which are amplified through microglia-astrocyte interactions.

Stroke injuries can induce the activation of microglia, which are generally classified into detrimental M1 and protective M2 subtypes (**Figure 2**). M1 microglia can secrete pro-inflammatory cytokines while M2 microglia can secrete anti-inflammatory cytokines. M2 microglia can stimulate neural stem/progenitor cell proliferation and neuronal differentiation in the ipsilateral subventricular zone through upregulation of TGFα expression levels. Studies have shown that in acute ischemia stroke, activated microglia predominantly express M2 phenotypic markers. However, there is a gradual shift to the M1 phenotype at around 1 week since the acute initiation of ischemia stroke, which persists for several weeks or even months. The sustained activation microglia is also thought to be associated with the onset and progression of neurodegenerative diseases (100). NF-κB, which is a key molecule in the microglia inflammatory pathway, induces activation and polarization of M1 microglia (101). Astrocytes can proliferate reactively after ischemic stroke. Liddelow et al. classified these astrocytes into the A1 and A2 subtypes, which are neurodamaging and neuroprotective, respectively (102). AHR plays an important role in activating microglia and activating astrocytes, which participate in the pro-inflammatory and anti-inflammatory processes, respectively. AHR inhibits the pro-inflammatory NF-κB signaling pathway while deletion of AHR or AHR ligands in microglia results in a dysregulated inflammatory response. Microglia and astrocytes intercommunicate with each other in many ways and may also be involved in the “gut-microbiota-brain” axis (103). Based on the fact that gut microbial metabolites can affect the CNS *via* the AHR-dependent signaling pathway, role of the commensal microbiota-mediated AHR signaling in the regulation of inflammation-promoting activity mediated by microglia and astrocytes has been investigated in recent years. Agonists derived from diet, gut microbiota and host metabolism can activate the AHR through the BBB. The AHR promotes TGFα expression in microglia, which acts on astrocytes and inhibits their pro-inflammatory activity. Further, AHR on microglia inhibits NF-κB-driven vascular endothelial growth factor B (VEGFB) expression, thereby promoting astrocytes to exert anti-inflammatory activity (104). Gut microbiota dysbiosis after stroke leads to abnormal tryptophan metabolism, and the decreased levels of AHR agonists may lead to enhanced neuroinflammation.

## AHR as a potential therapeutic target for treatment of ischemia stroke

As previously described, modulation of the AHR signaling may provide new therapeutic strategy to attenuate neuronal damage after acute ischemia stroke and prevent the development of post-stroke neurodegeneration, thereby improving the short- and long-term ischemia stroke prognosis. In the permanent MCAO mouse model, L-Kyn mediates ischemic neuronal injury as an endogenous activator of AHR (22). Therefore, pharmacological inhibition of the kynurenine pathway or activation of the AHR pathway in acute ischemia stroke might prevent neurological injury. On the one hand, early administration of TMF, an AHR antagonist, can be a simple approach for the treatment of acute ischemia stroke. On the other hand, synthesis of L-Kyn under the action of TDO as the primary pathway in ischemic brain tissue and inhibition of L-Kyn production by the TDO inhibitor compromises the activation of the AHR signaling, leading to reduced infarct volume. Interestingly, pharmacological blockade of IDO, another key enzyme in L-Kyn production, by the IDO inhibitor 1-MT, does not show a beneficial effect in reducing infarct size and improving neurological prognosis, despite increased IDO expression and activity in transient MCAO mouse model (105).

AHR can mediate the inflammatory response in glial cells of the CNS (106). Dietary and microbial metabolites, particularly tryptophan metabolites, have recently been shown to act as AHR activators and thus regulate microglia and astrocyte activity and neuroinflammation in the CNS (5, 94). These studies linked the gut microbiota to neurological inflammation in the brain *via* the AHR signaling pathway. In mice with autoimmune encephalomyelitis, the AHR signaling was activated in astrocytes, which was proven to limit the inflammatory response in astrocytes. Moreover, this anti-inflammatory response could become increasingly evident when dietary tryptophan was ingested by mice. To demonstrate that this effect is regulated by microbiota-mediated tryptophan metabolites, a broad-spectrum antibiotic-ampicillin was applied to the mice to clear their gut microbiota, followed by treatment of the mice with indirubin-3’-oxime, a microbial metabolite of tryptophan. As a result, AHR-mediated anti-inflammatory effects were observed (5), indicating the effect is indeed mediated by tryptophan metabolites. Indirubin-3’-oxime has also been shown to inhibit the inflammatory activation in microglia in the rat brain (107). *Lactobacillus* was found to be an important host probiotic, and its levels were reduced after cerebral ischemia in monkeys (23). There are also studies showing that *Lactobacillus* supplementation can improve cognitive function and mood and reduce aging-related inflammation in mice and rats (28, 108). *Lactobacillus casei subsp. casei 327* (327 strain) can indirectly promote colonic 5-HT synthesis (109). *Lactobacillus reuteri* can degrade tryptophan into indolic compounds, such as IAld, ILA, and IAA (74, 110). A decreasing trend in serum kynurenine: tryptophan ratios was observed in humans after 8 weeks of oral administration of *Lactobacillus johnsonii* (111). As an important source of essential amino acids, diet is considered to be an important factor in shaping microbial tryptophan metabolism. A recent study indicated that the microbial tryptophan degradation pathway could be weakened under a high-fat diet (112). In addition, increasing carbohydrate availability promotes intestinal serotonin synthesis (113). Thus, probiotic supplementation and a reasonable diet can theoretically improve ischemia stroke prognosis; however, whether it is indeed beneficial in post-stroke patients needs to be tested in future clinical trials. Ramos et al. showed that the function of AHR and its downstream signaling pathways are impaired in the elderly and AD patients (114). The role of AHR ligands in improving learning memory deficits was also confirmed in a mouse model (104). Activation of the AHR signaling pathway by endogenous ligands such as L-Kyn and 6-Formylindolo[3,2-b]carbazole (FICZ), or exogenous ligands such as diosmin and indole-3-carbinol, could increase the expression and enzymatic activity of neprilysin in amyloid precursor protein/presenilin 1 (APP/PS1) mice, and improve cognitive impairment effectively in these mice. Tryptophan metabolites, such as 5-hydroxy indole-acetic acid and kynurenic acid, could reduce cognitive impairment in mice and Aβ load in patients with mild cognitive impairment by activating AHR (115–118).

## Present shortcomings and future perspectives

New therapies, such as the application of recombinant thrombolytic tissue plasminogen activator (r-tPA) and intra-arterial thrombectomy, have been developed for the treatment of acute ischemia stroke (119). However, due to the narrow time windows and the limitations of endovascular treatment techniques, only a small number of patients with acute ischemia stroke can benefit from these new therapies. There are limited treatment options for patients with subacute and chronic ischemia stroke. In light of this, AHR can be used as a potential therapeutic target for the treatment of these patients (85). Inhibition of AHR signaling in acute ischemia stroke has the potential to benefit the patients by reducing pro-inflammatory gliosis and enhancing neurogenesis. In contrast, tryptophan metabolites, as the AHR ligands, can interact with microglia and astrocytes and prevent neurodegeneration. Supplementation of tryptophan metabolites, probiotics producing AHR agonists, and FMT from normal donors may be potential therapeutic strategies that can improve the prognosis of certain types of ischemia stroke. Delivering drugs to the brain directly has long been a major challenge in treating neurodegeneration, and thus these proposed strategies might overcome this barrier.

However, there is still a long way to go for researchers despite the substantial progress. Firstly, the composition and immunological characteristics of human gut microbiota are not completely the same as those of animals such as mice. Secondly, the effects of intestinal fungi and protozoa on tryptophan metabolism and severity of ischemia stroke are unclear. Whether there are other endogenous or exogenous AHR ligands besides tryptophan that have not been identified and whether there are any other endogenous inhibitors of the AHR pathway are unknown as well. Tryptophan can also directly be absorbed by the host gut and the complex interactions between intestinal flora, intestinal lumen tryptophan availability, and host tryptophan metabolism need further study. Thirdly, it requires validation that whether the results from animal studies could be used for the effective treatment of human diseases such as ischemia stroke. Developing a humanized mouse model might help explain the well-known differences regarding AHR between humans and mice. Finally, large-scale, highly controlled clinical studies are urgently needed to further validate the role of AHR in ischemia stroke development.

## Conclusion

The role of AHR and tryptophan metabolism in the communication between the gut microbiota and CNS has been increasingly well known. Tryptophan metabolism is directly or indirectly regulated by the gut microbiota and many tryptophan metabolites can act as endogenous AHR activators, activating AHR, which can further regulate neuroinflammation by interacting with microglia and astrocytes. Since many factors can affect the gut microbiota composition and metabolism, including diet, antibiotics, and probiotics, as well as FMT, the manipulation of the gut microbiota modulating tryptophan availability may be a therapeutic method for neuroinflammation after ischemia stroke. In conclusion, we argue that the AHR and tryptophan metabolism play an important role in ischemia stroke.

## Author contributions

Conception and design: SW and XF; writing of the manuscript: XF; final approval of the manuscript: HZ. All authors read and approved the final manuscript.

## Funding

This work was supported by the Construction Fund of Medical Key Disciplines of Hangzhou (discipline number OO20200485).

## Conflict of interest

The authors declare that the research was conducted in the absence of any commercial or financial relationships that could be construed as a potential conflict of interest.

## Publisher’s note

All claims expressed in this article are solely those of the authors and do not necessarily represent those of their affiliated organizations, or those of the publisher, the editors and the reviewers. Any product that may be evaluated in this article, or claim that may be made by its manufacturer, is not guaranteed or endorsed by the publisher.
